# Potato growth, photosynthesis, yield, and quality response to regulated deficit drip irrigation under film mulching in a cold and arid environment

**DOI:** 10.1038/s41598-021-95340-9

**Published:** 2021-08-05

**Authors:** Fuqiang Li, Haoliang Deng, Yucai Wang, Xuan Li, Xietian Chen, Lintao Liu, Hengjia Zhang

**Affiliations:** College of Water Conservancy and Hydropower Engineering, Gansu Agricultural University, Lanzhou, 730070 China

**Keywords:** Plant sciences, Plant stress responses

## Abstract

The effects of the amount and timing of regulated deficit drip irrigation under plastic film on potato (‘*Qingshu 168*’) growth, photosynthesis, yield, water use efficiency, and quality were examined from 2017 to 2019 in cold and arid northwestern China. In the four stages of potato growth (seedling, tuber initiation, tuber bulking, starch accumulation), eight treatments were designed, with a mild deficit was in treatments WD1 (seedling), WD2 (tuber initiation), WD3 (tuber bulking), and WD4 (starch accumulation); and a moderate deficit in WD5 (seedling), WD6 (tuber initiation), WD7 (tuber bulking), and WD8 (starch accumulation). The net photosynthetic rate, stomatal conductance, and transpiration rate decreased significantly under water deficit in the tuber formation and starch accumulation stages. Although water deficit reduced potato yields, a mild deficit in the seedling stage resulted in the highest yield and water use efficiency at 43,961.91 kg ha^−1^ and 8.67 kg m^−3^, respectively. The highest overall quality was in potatoes subjected to mild and moderate water deficit in the seedling stage. Principal component analysis identified mild water stress in the seedling stage as the optimum regulated deficit irrigation regime. The results of this study provide theoretical and technical references for efficient water-saving cultivation and industrialization of potato in northwestern China.

## Introduction

*Solanum tuberosum* L. is also known as the white or Irish potato and is rich in nutrients and starch. It is a popular food with the reputation of being a “lost treasure”, with medicinal and health care properties that include neutralizing the stomach, tonifying the spleen and qi, detoxifying, and relieving inflammation. Moreover, potato is the world’s fourth largest food crop after wheat, rice and corn^1^. The United Nations declared 2008 as the International Year of the Potato, which further demonstrated the status of the potato as the world’s number one non-cereal food in global food security. As of 2018, the world's potato planting area and total output were 1.8 × 10^7^ hm^2^ and 3.7 × 10^8^ t, respectively, while China's potato planting area and total output accounted for 27% and 24% of the world's total, respectively, ranking first in the world^2^. In recent years in the Hexi Corridor Oasis Irrigation Area in the Hexi Oasis, potato has been a popular crop because of its strong adaptability, simple cultivation, suitability for storage, ease of transportation, and long supply period. However, with scaled-up cultivation, irrigation systems that use water inefficiently have led to a continuous increase in demand for irrigation water. Therefore, the design of irrigation systems that increase irrigation water use efficiency is an important approach to optimize water use. Regulated deficit irrigation is an advanced irrigation technology that was first proposed and practically implemented by the Australian Sustainable Irrigation Agricultural Research Institute when studying methods to improve the productivity of densely planted orchards (mainly those with peach trees) in the 1970s^3^. With this technology, a crop experiences a regulated water deficit at different amounts according to the characteristics of water consumption for that crop, thereby saving water and increasing water use efficiency. Regulated deficit irrigation has been widely applied, including with corn^4,5^, tomatoes^6^, wheat^7^, onions^8,9^, *Isatis tinctoria*^10,11^, grapes^12,13^, persimmons^14^, cherries^15,16^, garlic^17^, watermelons^18^, and peppers^19^. The technology is effective at increasing yields and water conservation. In potato, Xue et al.^20^ studied the effects of regulated deficit irrigation with film in different growth stages on yield and water use efficiency in a desert oasis. They found that the highest indices of potato water sensitivity were in the starch accumulation stage, followed the tuber enlargement stage, with lower indices in the seedling and tuber formation stages. Thus, regulated deficit irrigation increased water use efficiency at the seedling and tuber formation stages while stabilizing yield. By comparison, the decrease in output caused by regulated deficit irrigation was from 28.30 to 44.32% in the starch accumulation stage and from 20.13 to 27.92% in the tuber enlargement stage. Martínez-Romero et al.^21^ found that a timely and moderate irrigation quota deficit improved potato yield, potato commodity rate, and water use efficiency in an experiment with regulated deficit irrigation in Basque, Spain. In addition, Zhang and Li^22^, Li et al.^23^, and Du et al.^24^ further developed the technology of regulated deficit drip irrigation under film for potato in arid areas by examining water use, growth, yield, and quality.

Various modifications of regulated deficit drip irrigation under film for potato in arid inland oases have been proposed in previous studies, which are highly instructive for the development of potato cultivation and water conservation. Current research primarily focuses on the effects of regulated deficit drip irrigation under film on potato yield and water use efficiency. However, less attention is paid to the effects on photosynthesis in potatoes, and reports on the effects on potato quality are rare. Soil and climatic conditions in different regions can affect the application of irrigation and cultivation technologies. Therefore, research to improve the cultivation system of regulated deficit drip irrigation under film in arid oasis areas is a long-term task. In this study, in the light loam soil of the Hexi Oasis, the effects of regulated deficit drip irrigation under film on potato growth, photosynthetic properties, quality, output, and water use efficiency were examined in a field experiment. The aim of this work was to study the sensitivity of each phenological stage of potato to different degrees of water deficit, for the application of regulated deficit irrigation strategies. The research results provide a theoretical basis for the establishment of a drought-resistant (water-saving) potato planting technology system.

## Results and analysis

### Effects of regulated deficit drip irrigation under film on the growth indices and tuber characteristics of potato

Irrigation with different levels of regulated deficit in different growth stages significantly affected the biological characteristics of potato (Table 1). Compared with that in CK, plant height in the treatments decreased, although the decrease in mild regulated deficit treatments (WD1, WD2, WD3, and WD4) was not significant (*P* > 0.05). In the moderate deficit treatments WD6 and WD7, plant height decreased significantly (*P* < 0.05). Stem diameter decreased in all treatments to various degrees but the smallest decrease was in WD1 and WD5 with the water deficit in the seedling stage. Compared with that in CK, the diameter decreased by 3.37% in WD1 and by 5.62% in WD5. However, when the deficit occurred in the tuber initiation and tuber bulking stages, the diameter decreased by 3.93–8.99% and by 6.74–10.64%, respectively, indicating that a water deficit in those two stages reduced potato stem growth.

**Table 1:** Indices of potato growth in conventional irrigation (CK) and regulated deficit drip irrigation treatments in 3 years and averaged across years.

Year	Treatment	Plant height (cm)	Stem diameter (cm)	Leaf area index	Longitudinal diameter (mm)	Cross diameter (mm)
2017	CK	141.62 ± 8.52a	1.74 ± 0.115a	1.25 ± 0.079a	83.89 ± 6.62a	65.62 ± 2.83ab
	WD1	139.55 ± 6.18ab	1.69 ± 0.146ab	1.19 ± 0.086ab	82.55 ± 5.97ab	65.74 ± 4.52a
	WD2	137.54 ± 7.74ab	1.62 ± 0.093b	1.12 ± 0.077bc	81.77 ± 7.06ab	63.88 ± 4.94ab
	WD3	139.39 ± 7.05ab	1.58 ± 0.109bc	1.07 ± 0.063c	80.96 ± 4.54ab	63.57 ± 5.46ab
	WD4	140.85 ± 11.60ab	1.48 ± 0.117cd	0.98 ± 0.041d	80.06 ± 6.24ab	61.34 ± 3.77bc
	WD5	136.01 ± 7.35ab	1.61 ± 0.125bc	1.13 ± 0.102b	82.03 ± 5.93ab	64.12 ± 4.32ab
	WD6	130.15 ± 9.24b	1.53 ± 0.073c	1.09 ± 0.077bc	81.01 ± 5.89ab	62.69 ± 4.79ab
	WD7	135.36 ± 8.68ab	1.36 ± 0.119d	1.02 ± 0.065cd	78.58 ± 4.68b	61.66 ± 5.31b
	WD8	137.52 ± 10.76ab	1.28 ± 0.084e	0.72 ± 0.040e	78.05 ± 5.64bc	57.62 ± 3.68c
2018	CK	144.3 ± 8.83a	1.93 ± 0.171a	1.76 ± 0.121ab	83.31 ± 4.25a	65.54 ± 2.84a
	WD1	140.5 ± 6.35ab	1.84 ± 0.154ab	1.66 ± 0.133bc	80.54 ± 5.02ab	63.81 ± 2.76ab
	WD2	136.3 ± 9.47ab	1.86 ± 0.162ab	1.51 ± 0.084c	79.86 ± 5.16ab	61.95 ± 2.13ab
	WD3	134.6 ± 8.51ab	1.88 ± 0.149ab	1.61 ± 0.153bc	78.55 ± 3.99ab	59.47 ± 2.57bc
	WD4	134.0 ± 9.13b	1.85 ± 0.138ab	1.84 ± 0.179ab	75.14 ± 3.28bc	57.18 ± 1.96bc
	WD5	133.0 ± 7.86bc	1.78 ± 0.085b	1.85 ± 0.095a	81.22 ± 4.11ab	61.88 ± 3.08ab
	WD6	126.0 ± 5.29bc	1.74 ± 0.107bc	1.76 ± 0.062ab	80.07 ± 4.53ab	60.35 ± 2.83b
	WD7	136.0 ± 8.15ab	1.81 ± 0.134ab	1.69 ± 0.105b	76.43 ± 4.35b	58.74 ± 3.61bc
	WD8	139.5 ± 11.17ab	1.86 ± 0.093ab	1.67 ± 0.735bc	72.69 ± 3.59bc	54.38 ± 3.37c
2019	CK	146.40 ± 6.92a	1.67 ± 0.130a	1.34 ± 0.101a	88.53 ± 5.71a	68.87 ± 5.60a
	WD1	145.10 ± 7.55ab	1.63 ± 0.148ab	1.29 ± 0.082ab	87.17 ± 6.19ab	68.09 ± 4.17ab
	WD2	143.80 ± 11.70ab	1.64 ± 0.117ab	1.25 ± 0.071b	86.59 ± 3.31ab	67.91 ± 5.28ab
	WD3	145.67 ± 10.92ab	1.53 ± 0.081b	1.24 ± 0.095bc	87.41 ± 7.89ab	64.38 ± 4.73b
	WD4	145.70 ± 11.58ab	1.60 ± 0.099ab	1.18 ± 0.064c	84.26 ± 6.08ab	62.93 ± 4.92bc
	WD5	140.37 ± 13.44ab	1.64 ± 0.132ab	1.19 ± 0.100bc	87.04 ± 5.32ab	67.44 ± 5.05ab
	WD6	133.07 ± 9.16bc	1.59 ± 0.118ab	0.99 ± 0.048de	85.59 ± 6.67ab	66.39 ± 4.77ab
	WD7	135.73 ± 10.07b	1.60 ± 0.095ab	1.23 ± 0.075bc	82.76 ± 6.08b	65.52 ± 2.86ab
	WD8	139.30 ± 11.39ab	1.45 ± 0.087c	1.01 ± 0.081d	75.54 ± 5.85c	59.48 ± 3.13c
Average	CK	144.11 ± 5.68a	1.78 ± 0.05a	1.45 ± 0.056a	85.24 ± 4.21a	66.68 ± 2.75a
	WD1	141.72 ± 4.25ab	1.72 ± 0.06ab	1.38 ± 0.03ab	83.42 ± 4.35ab	65.88 ± 2.61ab
	WD2	139.21 ± 3.97ab	1.71 ± 0.06ab	1.29 ± 0.06b	82.74 ± 4.16ab	64.58 ± 2.58ab
	WD3	139.89 ± 3.03ab	1.66 ± 0.05b	1.31 ± 0.04bc	82.31 ± 4.34ab	62.47 ± 1.93ab
	WD4	140.25 ± 4.11ab	1.64 ± 0.03bc	1.33 ± 0.08cd	79.82 ± 4.09ab	60.48 ± 1.88bc
	WD5	136.46 ± 3.23ab	1.68 ± 0.07ab	1.39 ± 0.04bc	83.43 ± 4.41ab	64.48 ± 3.04ab
	WD6	129.74 ± 3.05bc	1.62 ± 0.05b	1.28 ± 0.03d	82.22 ± 4.08ab	63.14 ± 2.95ab
	WD7	135.7 ± 4.86b	1.59 ± 0.05c	1.31 ± 0.07c	79.26 ± 4.12b	61.97 ± 2.43b
	WD8	138.77 ± 4.27ab	1.53 ± 0.08d	1.13 ± 0.03e	75.43 ± 3.94bc	57.16 ± 2.24c
ANOVA	Treatment (T)	*	**	**	*	*
	Year (Y)	ns	ns	ns	ns	ns
	T × Y	ns	ns	ns	ns	ns

Diameters are those of tubers.

Different lowercase letters within a column for a year or the average indicate significant differences among treatments (*P* < 0.05). The irrigation treatments were conventional irrigation (CK) and mild or moderate water deficit during each of four stages of potato growth. Mild deficit was in treatments WD1 (seedling), WD2 (tuber initiation), WD3 (tuber bulking), and WD4 (starch accumulation); moderate deficit was in treatments WD5 (seedling), WD6 (tuber initiation), WD7 (tuber bulking), and WD8 (starch accumulation). Values followed by the same lowercase letters within each year are not significantly different at the *P* < 0.05 level. *, **, and *** are significant at the *P* < 0.05, 0.01, and 0.001 levels, respectively.

*ns* not significant.

Compared with CK, all treatments caused the leaf area index to decrease to varying degrees, ranging from 4.14 to 22.07%. The greatest decreases in leaf area index were in WD6 and WD8, with significant decreases of 11.72% and 22.07%, respectively. The leaf area index was least affected in WD5, with a decrease of only 4.14%. This result showed that mild water deficit at the seedling stage did not significantly affect leaf growth. By contrast, water deficit in the other stages hindered leaf growth. Both the longitudinal and transverse lengths of the potato tubers decreased, to different degrees. The greatest effect was in WD8, with significant decreases ranging from 11.51 to 14.28%. In WD1, WD2, WD5, and WD6, the decreases in both diameters were slight and not significant.

### Potato leaf photosynthetic characteristics

#### Net photosynthetic rate

**Figure 1:**
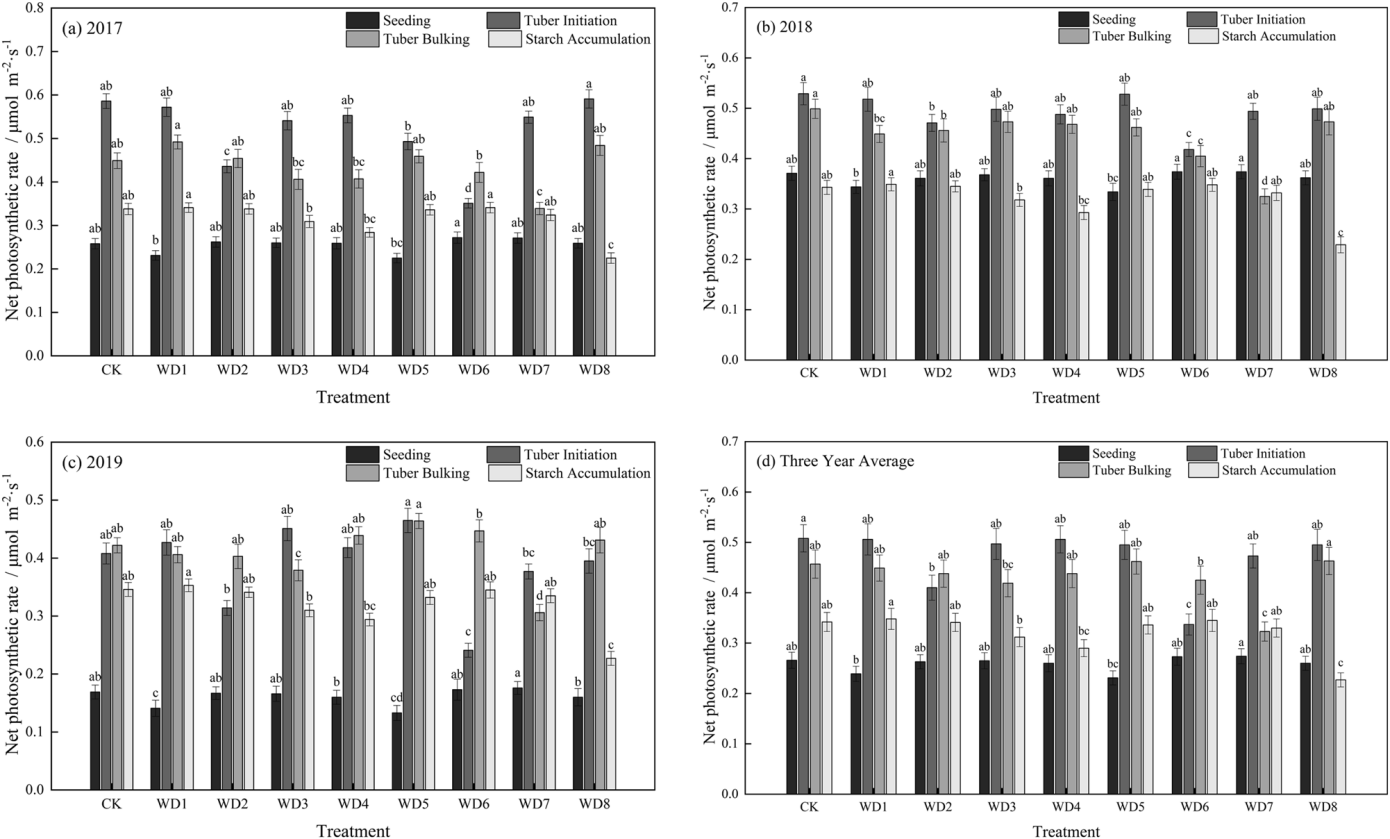
The effect of regulated deficit drip irrigation under film on the net photosynthetic rate (µmol m^−2^ s^−1^) of potato leaf. The irrigation treatments were conventional irrigation (CK) and mild or moderate water deficit during each of four stages of potato growth. Mild deficit was in treatments WD1 (seedling), WD2 (tuber initiation), WD3 (tuber bulking), and WD4 (starch accumulation); moderate deficit was in treatments WD5 (seedling), WD6 (tuber initiation), WD7 (tuber bulking), and WD8 (starch accumulation).

The net photosynthetic rate (Pn) of potato leaf from seedling to starch accumulation stage increased first and then decreased, showing a single-peak in CK and all treatments (Fig. 1). Mild regulated deficit treatments did not significantly affect the rate in the seedling stage. In the moderate deficit treatment WD5, the Pn decreased by 13.14% compared that with in CK, although the decrease was not significant. In the tuber initiation stage, the rate decreased significantly to 0.410 μmol m^−2^ s^−1^ in WD2 (mild deficit) and to 0.337 μmol m^−2^ s^−1^ in WD6 (moderate deficit), decreases of 19.28% (WD2) and 33.70% (WD6) compared with that in CK. The Pn was not significantly affected in the other treatments in this stage.

In the tuber bulking stage, the Pn decreased by 8.31% in WD3 (mild deficit) and by 29.37% in WD7 (moderate deficit), compared with that in CK. The compensation effect in WD2 after rehydration was greater than that in WD6, but neither rate was significantly different from that in CK. The Pn did not decrease from the tuber initiation stage to the tuber expansion stage, and the mean value in the bulking stage was between 0.323 and 0.463 μmol m^−2^ s^−1^. In the starch accumulation stage, the absolute value of the Pn was lower than that at tuber initiation and expansion stages, with a mean value between 0.227 and 0.348 μmol m^−2^ s^−1^. However, the Pn was significantly higher than that in the seedling stage. The Pn decreased by 15.12% in WD4 (mild deficit) and by 33.46% in WD8 (moderate deficit), compared with that in CK, indicating that a regulated deficit at the starch accumulation stage negatively affected the net photosynthetic rate.

#### Stomatal conductance

**Figure 2:**
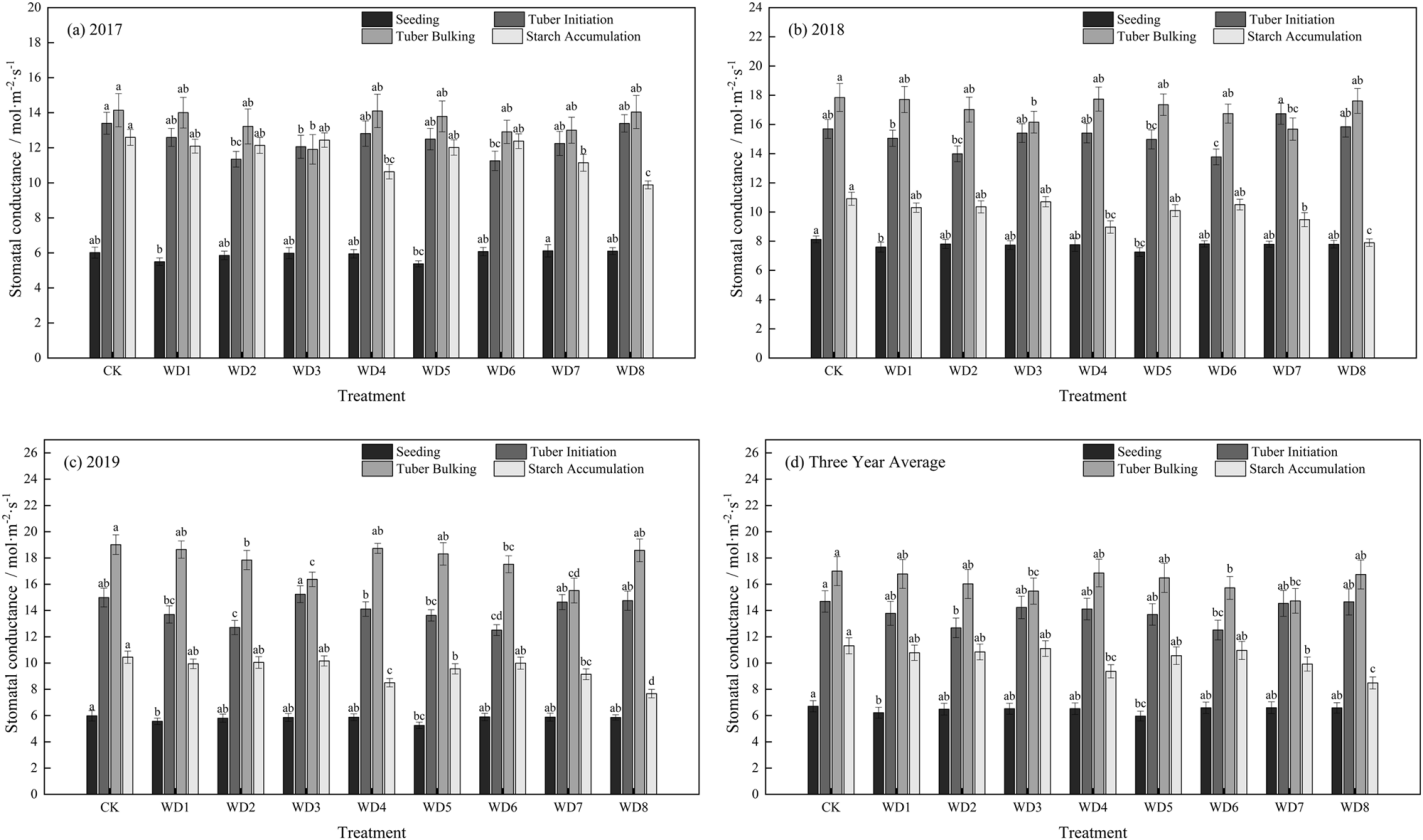
The effect of regulated deficit drip irrigation under film on the stomatal conductance (mol m^−2^ s^−1^) of potato leaf. The irrigation treatments were conventional irrigation (CK) and mild or moderate water deficit during each of four stages of potato growth. Mild deficit was in treatments WD1 (seedling), WD2 (tuber initiation), WD3 (tuber bulking), and WD4 (starch accumulation); moderate deficit was in treatments WD5 (seedling), WD6 (tuber initiation), WD7 (tuber bulking), and WD8 (starch accumulation).

Throughout the growth period, the stomatal conductance (Gs) increased successively from the seedling stage to the tuber initiation stage and then tuber bulking stage; however, the Gs decreased at the starch accumulation stage. There were differences in the range of decrease among the treatments (Fig. 2). The lowest Gs was in the seedling stage, ranging from 5.96 to 6.71 mol m^−2^ s^−1^. In WD1 (mild deficit) and WD5 (moderate deficit), Gs decreased significantly by 7.29% and 11.09%, respectively, compared with that in CK. The Gs in the seedling stage was not affected in the other treatments. The Gs increased in the tuber initiation stage in all treatments except WD2 and WD6, with values increasing to between 12.68 and 14.70 mol m^−2^ s^−1^. The Gs in WD2 decreased by 13.70% and that in WD6 decreased by 14.83%. In the tuber bulking stage, Gs increased to the highest levels of the entire growth period, ranging from 14.73 to 17.00 mmol m^−2^ s^−1^, although it was lower in regulated deficit treatments than in CK. Compared with that in CK, the Gs decreased significantly in WD3 by 8.96% and in WD7 by 13.34%, which showed that regulated deficit in the tuber initiation and expansion stages did not favor stomatal opening. The Gs decreased significantly from the tuber bulking stage to the starch accumulation stage. With sufficient irrigation in this period, Gs was not significantly different from that in CK, whereas in WD4 (mild deficit) and WD8 (moderate deficit), it decreased significantly by 17.22% and 25.05%, respectively. This result demonstrated that regulated deficit during starch accumulation affected stomatal conductance.

#### Transpiration rate

**Figure 3:**
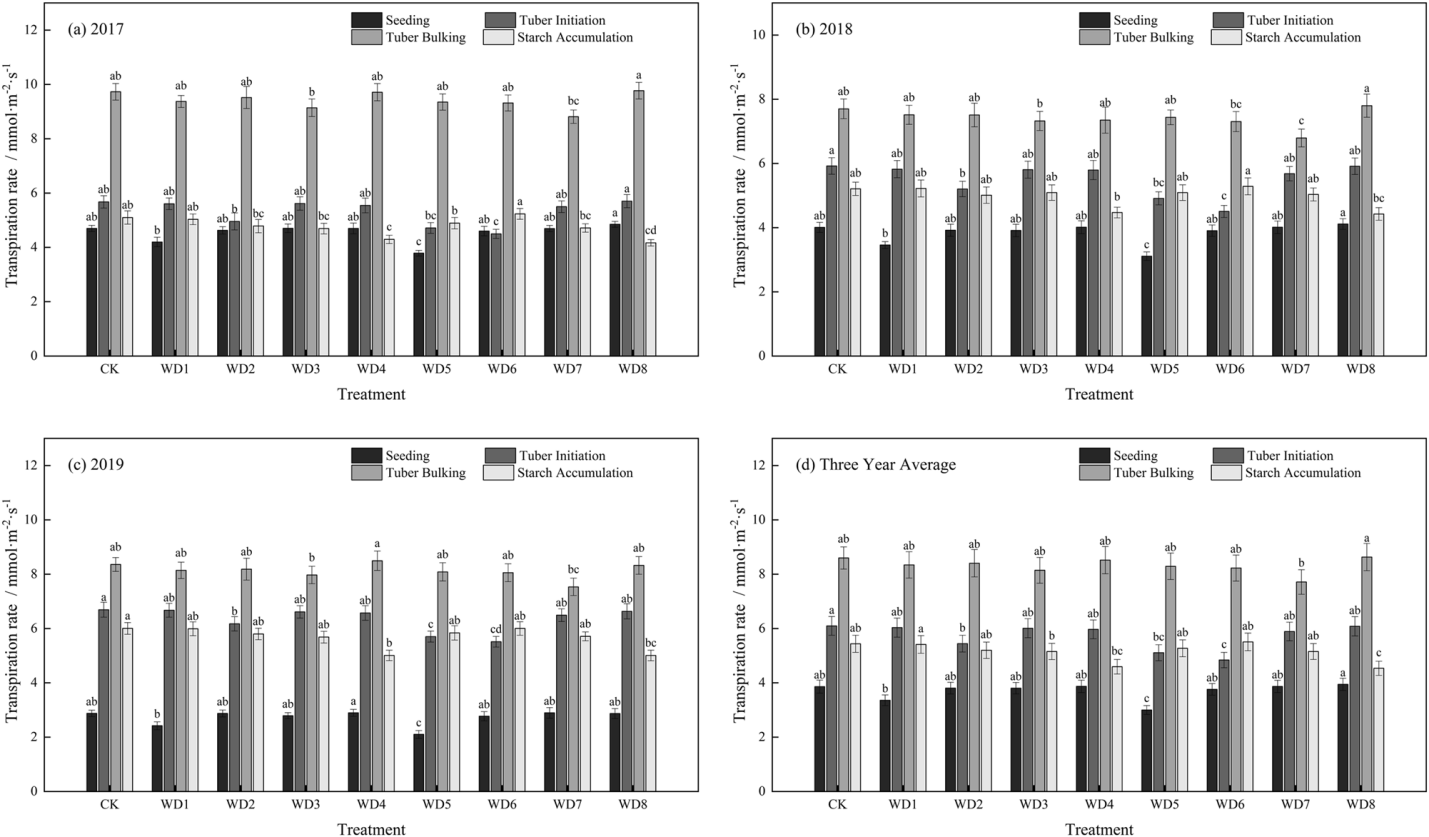
The effect of regulated deficit drip irrigation under film on the transpiration rate (mmol m^−2^ s^−1^) of potato leaf. The irrigation treatments were conventional irrigation (CK) and mild or moderate water deficit during each of four stages of potato growth. Mild deficit was in treatments WD1 (seedling), WD2 (tuber initiation), WD3 (tuber bulking), and WD4 (starch accumulation); moderate deficit was in treatments WD5 (seedling), WD6 (tuber initiation), WD7 (tuber bulking), and WD8 (starch accumulation).

In all treatments, the transpiration rate (Tr) first increased as growth progressed and then decreased, with a peak at the tuber expansion stage (Fig. 3). In the seedling stage, the Tr decreased by 13.01% in WD1 (mild deficit) and by 22.29% in WD5 (moderate deficit), compared with that in CK. In the tuber initiation stage, the Tr was 5.45 μmol mol^−1^ s^−1^ in WD2 (mild deficit) and 4.84 μmol mol^−1^ s^−1^ in WD6 (moderate deficit), decreasing by 10.68% in WD2 and by 20.63% in WD6 compared with that in CK.

In the tuber bulking stage, the Tr in WD1, WD2, WD5, and WD6 was not significantly different from that in CK after rehydration, showing evidence of a compensation effect. Compared with the tuber initiation stage, the Tr showed a large increase in all treatments. The Tr in WD7 (moderate deficit) decreased significantly by 10.28% compared with that in CK, whereas in WD3 (mild deficit), the Tr decreased by 5.25%, but it was not significantly different from that in CK. In the tuber initiation and tuber bulking stages, the Tr was higher than that in the seedling stage. In the starch accumulation stage, the Tr was 4.59 μmol mol^−1^ s^−1^ in WD4 (mild deficit) and 4.53 μmol mol^−1^ s^−1^ in WD8 (moderate deficit), decreasing by 15.58% in WD4 and by 16.64% in WD8 compared with that in CK, although the decreases were not significant. After rehydration, the Tr in WD3 and WD7 decreased, but it was not significantly different from that in CK.

#### Tuber yield and its components

Regulated deficit in all growth stages significantly affected water consumption, yield, water use efficiency, and irrigation water use efficiency (Table 2). Water deficit in all growth stages significantly decreased water consumption by potato. Compared with 5837 m^3^ ha^−1^ in CK, water consumption decreased to 4977 m^3^ ha^−1^ in WD3: 4952 m^3^ ha^−1^ in WD5: 4769 m^3^ ha^−1^ in WD6, and 4724 m^3^ ha^−1^ in WD8, which were decreases of 14.73% (WD3), 15.16% (WD5), 18.30% (WD6), and 19.08% (WD8) compared with that in CK. In WD2 (mild deficit) and WD7 (moderate deficit), the decrease was slight. The regulated deficit treatments caused decreases in potato yield to different degrees. The water deficit in the seedling stage in WD1 caused the smallest decrease in yield to 43,962 kg ha^−1^, a decrease of only 4.50% compared with that in CK. Water deficit in the tuber bulking stage in WD7 (moderate deficit) caused the greatest decrease in yield to 33,835 kg ha^−1^, a decrease of 26.50% compared with that in CK. The decreases in yield under moderate regulated deficit treatment were greater than those under mild treatment, indicating that the larger decrease in irrigation had a greater negative effect on potato yield. Affected by yield, the output values showed similar trends by treatment. Output value per cubic water was the lowest in WD7 (moderate deficit), decreasing by 15.56% compared with that in CK. By contrast, the values increased in the other treatments by 0.07–50.93%, compared with that in CK.

**Table 2:** Potato yield, output value, and water use in conventional irrigation (CK) and regulated water deficit drip irrigation treatments in 3 years and averaged across years.

Years	Treatment	Rainfall	Irrigation volume	Water consumption	Yield	Output value	Unilateral aquatic product value	Water use efficiency	Irrigation water use efficiency
		(m^3^ ha^−1^)	(m^3^ ha^−1^)	(m^3^ ha^−1^)	(kg ha^−1^)	(yuan ha^−1^)	(yuan m^−3^)	(kg m^−3^)	(kg m^−3^)
2017	CK	3017.5	2574.50 ± 122.78a	6462.20 ± 248.58a	58,160.00 ± 1632.29a	75,608.03 ± 1942.68a	29.37 ± 1.45 cd	9.00 ± 0.51b	22.59 ± 1.13 cd
	WD1	3017.5	1829.40 ± 84.79c	4883.70 ± 134.85 cd	50,400.20 ± 1316.02b	65,520.25 ± 1965.67b	35.81 ± 1.57bc	10.32 ± 0.49ab	27.55 ± 1.08bc
	WD2	3017.5	2074.50 ± 102.53b	5096.00 ± 203.84bc	45,099.90 ± 1308.69c	58,629.90 ± 1775.04c	28.26 ± 1.13 cd	8.85 ± 0.54bc	21.74 ± 1.02 cd
	WD3	3017.5	1741.70 ± 85.07 cd	4761.50 ± 191.22 cd	40,234.40 ± 1609.38d	52,304.71 ± 1415.69d	30.03 ± 1.51c	8.45 ± 0.47bc	23.10 ± 1.16 cd
	WD4	3017.5	1895.70 ± 91.26bc	4912.10 ± 194.65c	49,858.30 ± 1449.36bc	64,815.73 ± 1947.16bc	34.19 ± 1.59bc	10.15 ± 0.54ab	26.30 ± 1.13c
	WD5	3017.5	1479.60 ± 73.98d	4495.20 ± 181.46 cd	48,548.10 ± 1419.24bc	63,112.49 ± 1837.93bc	42.65 ± 2.05a	10.80 ± 0.65a	32.81 ± 1.46bc
	WD6	3017.5	1884.90 ± 92.44bc	4904.00 ± 166.85 cd	42,223.50 ± 1488.69 cd	54,890.52 ± 1567.46 cd	29.12 ± 1.32 cd	8.61 ± 0.42bc	22.40 ± 1.12a
	WD7	3017.5	2484.30 ± 115.42ab	5502.70 ± 218.22b	35,327.20 ± 1423.57e	45,925.33 ± 1377.76e	18.49 ± 0.92d	6.42 ± 0.39c	14.22 ± 1.64 cd
	WD8	3017.5	1371.30 ± 65.86de	3682.30 ± 124.79d	38,590.70 ± 1354.62de	50,167.92 ± 1567.03de	36.58 ± 1.47b	10.48 ± 0.58ab	28.14 ± 1.37d
2018	CK	1974	2805.24 ± 126.04a	5520.10 ± 208.27a	35,317.71 ± 1407.84a	45,913.02 ± 1339.77a	16.37 ± 0.83c	6.39 ± 0.33bc	12.59 ± 0.56b
	WD1	1974	2540.72 ± 125.62b	4859.10 ± 163.92bc	31,913.54 ± 1254.26bc	41,487.60 ± 1241.83bc	16.33 ± 0.65cd	6.57 ± 0.27bc	12.56 ± 0.62c
	WD2	1974	2530.99 ± 117.49bc	4465.80 ± 178.63c	34,852.08 ± 1332.94ab	45,307.70 ± 1323.19ab	17.90 ± 0.58bc	7.80 ± 0.41ab	13.77 ± 0.48cd
	WD3	1974	2753.43 ± 131.76ab	4807.40 ± 186.22bc	29,747.92 ± 1116.89c	38,672.30 ± 1109.53c	14.05 ± 0.72d	6.18 ± 0.37c	10.80 ± 0.45bc
	WD4	1974	2730.44 ± 122.35ab	5252.72 ± 208.11ab	33,619.79 ± 1379.44ab	43,705.73 ± 1311.17ab	16.01 ± 0.81cd	6.40 ± 0.28bc	12.31 ± 0.57d
	WD5	1974	2321.73 ± 108.16c	5010.40 ± 194.14b	32,508.33 ± 1300.21bc	42,260.83 ± 1224.65bc	18.20 ± 0.77b	6.49 ± 0.34bc	14.00 ± 0.63cd
	WD6	1974	2108.91 ± 104.72d	4110.80 ± 162.43d	32,879.17 ± 1219.66b	42,742.92 ± 1276.82b	20.27 ± 1.01a	7.99 ± 0.57a	15.59 ± 0.77b
	WD7	1974	2072.00 ± 101.33de	4227.90 ± 159.12cd	28,633.33 ± 1132.45cd	37,223.33 ± 1116.05cd	17.96 ± 0.69bc	6.77 ± 0.42b	13.82 ± 0.61a
	WD8	1974	2460.65 ± 122.53bc	5224.00 ± 206.98ab	34,050.00 ± 1263.54ab	44,265.00 ± 1237.94ab	17.99 ± 0.85bc	6.52 ± 0.31bc	13.84 ± 0.59bc
2019	CK	3051.3	2337.70 ± 116.88a	5529.30 ± 217.85a	44,621.97 ± 1478.61ab	58,008.57 ± 1571.25ab	33.70 ± 1.56e	8.07 ± 0.48b	19.09 ± 0.75cd
	WD1	3051.3	2128.90 ± 104.65ab	5438.40 ± 213.56ab	49,572.00 ± 1829.84a	64,443.60 ± 1908.33a	30.27 ± 1.17f.	9.12 ± 0.52a	23.29 ± 1.14b
	WD2	3051.3	2042.90 ± 102.11b	5463.00 ± 212.85ab	43,098.02 ± 1623.92bc	56,027.42 ± 1626.85bc	49.63 ± 2.15b	7.89 ± 0.34bc	21.10 ± 1.02bc
	WD3	3051.3	1807.80 ± 88.93c	5362.40 ± 214.46ab	39,309.81 ± 1439.85bc	51,102.75 ± 1525.08bc	35.42 ± 1.77de	7.33 ± 0.29bc	21.74 ± 1.08bc
	WD4	3051.3	1972.10 ± 95.86bc	5422.50 ± 215.83ab	43,308.67 ± 1632.68b	56,301.27 ± 1618.03b	69.70 ± 3.41a	7.99 ± 0.37bc	21.96 ± 0.98bc
	WD5	3051.3	1695.30 ± 84.71cd	5350.50 ± 212.04ab	40,364.17 ± 1614.57bc	52,473.42 ± 1574.26bc	38.24 ± 1.08d	7.54 ± 0.44bc	23.81 ± 1.15ab
	WD6	3051.3	1793.80 ± 85.69cd	5292.60 ± 207.14b	37,281.21 ± 1424.91cd	48,465.57 ± 1435.17cd	44.25 ± 2.21c	7.04 ± 0.39cd	20.78 ± 0.96c
	WD7	3051.3	1983.30 ± 91.65bc	5188.30 ± 205.36c	37,544.06 ± 1451.76c	48,807.28 ± 1421.64c	30.62 ± 1.35ef	7.24 ± 0.41c	18.93 ± 0.94cd
	WD8	3051.3	1521.50 ± 75.67d	5264.60 ± 210.59b	40,158.12 ± 1532.48bc	52,205.56 ± 1518.53bc	44.12 ± 2.06cd	7.63 ± 0.46bc	26.39 ± 1.21a
Average	CK	2680.93	2572.48 ± 153.12a	5837.20 ± 232.35a	46,033.23 ± 1276.38a	59,843.21 ± 1935.86a	26.48 ± 1.46cd	7.82 ± 0.32ab	18.09 ± 1.04cd
	WD1	2680.93	2166.34 ± 120.04bc	5060.40 ± 276.33bc	43,961.91 ± 1462.71ab	57,150.48 ± 1842.08ab	27.47 ± 1.28c	8.67 ± 0.25a	21.13 ± 1.17b
	WD2	2680.93	2216.13 ± 127.96b	5008.27 ± 249.69bc	41,016.67 ± 1201.57b	53,321.67 ± 1993.21b	31.93 ± 1.15bc	8.18 ± 0.46ab	18.87 ± 1.09c
	WD3	2680.93	2100.98 ± 123.05bc	4977.10 ± 226.87bc	36,430.71 ± 1582.22c	47,359.92 ± 1824.52c	26.50 ± 1.37cd	7.32 ± 0.32b	18.55 ± 1.05cd
	WD4	2680.93	2199.41 ± 102.33bc	5195.77 ± 274.51b	42,262.25 ± 1357.54ab	54,940.91 ± 1692.46ab	39.97 ± 2.25a	8.18 ± 0.17ab	20.19 ± 1.13bc
	WD5	2680.93	1832.21 ± 112.57cd	4952.03 ± 218.64bc	40,473.53 ± 1428.18bc	52,615.58 ± 1653.38bc	33.03 ± 1.68b	8.28 ± 0.38ab	23.54 ± 1.54a
	WD6	2680.93	1929.20 ± 122.35c	4769.13 ± 232.98bc	37,461.29 ± 1302.16bc	48,699.67 ± 1922.06bc	31.21 ± 1.76bc	7.88 ± 0.24ab	19.59 ± 0.65bc
	WD7	2680.93	2179.87 ± 107.06bc	4972.97 ± 214.17bc	33,834.86 ± 1557.96cd	43,985.31 ± 1769.23cd	22.36 ± 1.29d	6.81 ± 0.33bc	15.66 ± 1.27d
	WD8	2680.93	1784.48 ± 103.44cd	4723.63 ± 195.17bc	37,599.61 ± 1321.47bc	48,879.49 ± 1853.11bc	32.90 ± 1.03bc	8.21 ± 0.42ab	22.79 ± 1.05ab

The irrigation treatments were conventional irrigation (CK) and mild or moderate water deficit during each of four stages of potato growth. Mild deficit was in treatments WD1 (seedling), WD2 (tuber initiation), WD3 (tuber bulking), and WD4 (starch accumulation); moderate deficit was in treatments WD5 (seedling), WD6 (tuber initiation), WD7 (tuber bulking), and WD8 (starch accumulation).

#### Effect of regulated deficit drip irrigation under film on potato quality

Regulated deficit treatment had different effects on the characteristics of potato quality (Table 3). Compared with that in CK, deficient irrigation in the seedling stage did not significantly affect total sugar content in potato. However, in the other growth stages, regulated deficit reduced sugar content by 6.55–44.64%, and the greatest reduction was in WD8 with the deficit at the starch accumulation stage. With the deficit in the seedling stage, the protein content in WD5 (moderate deficit) was 2.18 mg g^−1^, an increase of 0.77% compared with that in CK. The protein contents decreased by 4.46–32.46% in the other treatments. The greatest reductions compared with the protein content in CK were in WD3 (18.31%), WD7 (21.85%), and WD8 (32.46%). With the deficit in the seedling stage, the starch content reached 36.06% in WD1 (mild deficit), a 3.34% increase compared with that in CK. In the other mild deficit treatments, although the starch content decreased, it was not significantly different from that in CK. In the moderate deficit treatments, the starch content decreased significantly by 10.66% in WD6, by 20.41% in WD7, and by 27.55% in WD8. With the deficit in the seedling stage, vitamin C and calcium contents decreased significantly by 9.21% in WD1 (mild deficit), compared with the contents in CK. However, the vitamin C content increased in the other mild and moderate deficit treatments, with the values increasing by 11.55–55.35% compared with that in CK. In addition, the vitamin C content tended to increase with the delay in deficit treatment, and the greatest increase was in WD8, with the content increasing significantly by 55.35% compared with that in CK.

**Table 3:** Indices of potato quality at harvest in conventional irrigation (CK) and regulated deficit drip irrigation treatments in 3 years and averaged across years.

Year	Treatment	Total sugar	Protein	Starch	Vitamin C
		(%)	(mg g^−1^)	(%)	(mg·100 g^−1^)
2017	CK	0.62 ± 0.03a	2.31 ± 0.06b	41.42 ± 1.54ab	15.77 ± 0.65de
	WD1	0.59 ± 0.02ab	2.29 ± 0.08bc	43.01 ± 1.62a	14.26 ± 0.71e
	WD2	0.54 ± 0.03bc	2.14 ± 0.05bc	40.95 ± 1.66ab	17.02 ± 0.81d
	WD3	0.43 ± 0.02de	1.88 ± 0.08c	38.36 ± 1.45bc	18.53 ± 0.94cd
	WD4	0.48 ± 0.02cd	2.17 ± 0.07bc	37.79 ± 1.46bc	20.15 ± 1.01bc
	WD5	0.57 ± 0.03b	2.54 ± 0.12a	38.54 ± 1.54b	19.07 ± 0.92c
	WD6	0.51 ± 0.03c	2.27 ± 0.06bc	36.05 ± 1.37bc	21.53 ± 1.02b
	WD7	0.44 ± 0.02d	1.72 ± 0.07d	30.26 ± 1.21c	21.97 ± 1.06ab
	WD8	0.36 ± 0.02e	1.54 ± 0.05e	27.93 ± 1.13cd	23.49 ± 1.18a
2018	CK	0.32 ± 0.03b	1.93 ± 0.04ab	19.33 ± 0.85ab	12.14 ± 0.52e
	WD1	0.37 ± 0.01a	1.99 ± 0.05a	19.40 ± 0.97a	11.57 ± 0.31ef
	WD2	0.22 ± 0.03d	1.9 ± 0.44ab	18.92 ± 0.67ab	13.35 ± 0.46d
	WD3	0.31 ± 0.02bc	1.68 ± 0.01bc	18.18 ± 0.73bc	14.05 ± 0.27cd
	WD4	0.30 ± 0.02c	1.91 ± 0.07ab	19.02 ± 0.91ab	14.92 ± 0.42c
	WD5	0.29 ± 0.03cd	1.59 ± 0.05c	18.37 ± 0.55b	13.31 ± 0.39de
	WD6	0.21 ± 0.01de	1.46 ± 0.05d	18.15 ± 0.63bc	16.97 ± 0.55b
	WD7	0.30 ± 0.02c	1.71 ± 0.06b	19.30 ± 0.79ab	15.63 ± 0.49bc
	WD8	0.17 ± 0.02e	1.38 ± 0.02e	17.50 ± 0.61bc	19.38 ± 0.92a
2019	CK	0.68 ± 0.04a	2.29 ± 0.08ab	42.68 ± 1.97ab	15.01 ± 0.81e
	WD1	0.68 ± 0.03a	2.26 ± 0.05b	44.39 ± 1.88a	13.35 ± 0.52f.
	WD2	0.59 ± 0.04b	2.16 ± 0.07bc	41.87 ± 1.83ab	16.84 ± 0.65d
	WD3	0.48 ± 0.02c	1.82 ± 0.04c	39.79 ± 1.73bc	18.41 ± 1.14cd
	WD4	0.47 ± 0.03cd	2.10 ± 0.04bc	38.91 ± 1.84bc	19.79 ± 1.07bc
	WD5	0.64 ± 0.03ab	2.48 ± 0.07a	39.87 ± 1.79b	18.97 ± 0.93c
	WD6	0.56 ± 0.02bc	2.20 ± 0.06bc	37.69 ± 1.63bc	21.06 ± 0.99b
	WD7	0.48 ± 0.02c	1.69 ± 0.03cd	32.01 ± 1.55c	21.72 ± 1.21ab
	WD8	0.38 ± 0.01d	1.51 ± 0.05d	29.17 ± 1.47d	23.05 ± 1.19a
Average	CK	0.56 ± 0.03ab	2.17 ± 0.07ab	34.89 ± 1.17ab	14.05 ± 0.58d
	WD1	0.58 ± 0.02a	2.17 ± 0.11ab	36.06 ± 1.05a	12.76 ± 0.64e
	WD2	0.47 ± 0.02c	2.07 ± 0.05ab	34.22 ± 0.96ab	15.67 ± 0.59cd
	WD3	0.42 ± 0.03d	1.77 ± 0.06c	32.59 ± 1.21bc	16.95 ± 0.67cd
	WD4	0.41 ± 0.01de	2.03 ± 0.05ab	32.28 ± 0.89bc	18.17 ± 0.61bc
	WD5	0.52 ± 0.04b	2.18 ± 0.13a	32.70 ± 0.76b	17.08 ± 0.52c
	WD6	0.44 ± 0.02cd	1.95 ± 0.08b	31.17 ± 1.03bc	19.69 ± 0.87b
	WD7	0.42 ± 0.03de	1.69 ± 0.04cd	27.77 ± 1.25c	19.69 ± 0.86bc
	WD8	0.31 ± 0.02e	1.46 ± 0.05d	25.28 ± 0.94cd	21.83 ± 1.17a
ANOVA	Treatment (T)	**	**	**	**
	Year (Y)	ns	ns	ns	ns
	T × Y	ns	ns	ns	ns

Different lowercase letters within a column for a year or the average indicate significant differences among treatments (*P* < 0.05). The irrigation treatments were conventional irrigation (CK) and mild or moderate water deficit during each of four stages of potato growth. Mild deficit was in treatments WD1 (seedling), WD2 (tuber initiation), WD3 (tuber bulking), and WD4 (starch accumulation); moderate deficit was in treatments WD5 (seedling), WD6 (tuber initiation), WD7 (tuber bulking), and WD8 (starch accumulation).Values followed by the same lowercase letters within each year are not significantly different at the *P* < 0.05 level. *, **, and ***are significant at the *P* < 0.05, 0.01, and 0.001 levels, respectively.

*ns* not significant.

#### Water use efficiency and irrigation water use efficiency

With water deficit in the starch accumulation stage, water use efficiency increased most significantly in WD4 by 13.49 yuan m^−3^, an increase of 50.93% compared with that in CK. Water use efficiency was the highest in WD1 at 8.67 kg m^−3^, followed by WD5, WD4, and WD8, representing increases of 10.87% (WD1), 5.84% (WD5), 4.60% (WD4), and 4.99% (WD8) compared with that in CK. By contrast, WUE decreased by 6.39% in WD3 and by 12.92% in WD7 (6.81 kg m^−3^) compared with that in CK. The lowest IWUE was in WD7 at only 15.66 kg m^−3^, a decrease of 13.45% compared with that in CK. By contrast, IWUE increased in the other treatments by 2.52–30.13%. The highest IWUE was in WD5 at 23.54 kg m^−3^, followed by WD8 at 22.79 kg m^−3^, increases of 30.13% and 25.98%, respectively, compared with that in CK.

#### Comprehensive evaluation of different irrigation deficit methods

We calculated the correlation matrix of 11 beneficial evaluation indices of the regulated deficit irrigation methods in the *Hexi* Oasis (Tables 4, 5, 6). Feature analysis of the matrix showed that the first five major components (comprehensive indices) had an accumulated contribution of 99.57% to the evaluation equation. Thus, we established the comprehensive formulas based on the first five indices: yield, WUE, IWUE, output value per cubic water, and output value:

$$ \begin{aligned} {\text{Y}}1 = & 0.3465{\text{X}}_{1} + 0.238{\text{X}}_{2} + 0.0638{\text{X}}_{3} + 0.0621{\text{X}}_{4} + 0.3465{\text{X}}_{5} + 0.3202{\text{X}}_{6} \\ & + \;0.3131{\text{X}}_{7} + 0.3501{\text{X}}_{8} - 0.341{\text{X}}_{9} + 0.3611{\text{X}}_{10} - 0.3542{\text{X}}_{11} , \\ {\text{Y}}2 = & 0.0717{\text{X}}_{1} + 0.362{\text{X}}_{2} + 0.6094{\text{X}}_{3} + 0.61{\text{X}}_{4} + 0.0717{\text{X}}_{5} - 0.2455{\text{X}}_{6} \\ & + \;0.0025{\text{X}}_{7} - 0.1024{\text{X}}_{8} + 0.2{\text{X}}_{9} - 0.0627{\text{X}}_{10} + 0.0229{\text{X}}_{11} , \\ {\text{Y}}3 = & - 0.2953{\text{X}}_{1} - 0.4626{\text{X}}_{2} + 0.2021{\text{X}}_{3} + 0.2009{\text{X}}_{4} - 0.2953{\text{X}}_{5} + 0.2318{\text{X}}_{6} \\ & + \;0.6051{\text{X}}_{7} + 0.2362{\text{X}}_{8} + 0.2301{\text{X}}_{9} + 0.0246{\text{X}}_{10} - 0.0354{\text{X}}_{11} , \\ {\text{Y}}4 = & 0.2945{\text{X}}_{1} + 0.1369{\text{X}}_{2} - 0.0846{\text{X}}_{3} - 0.0831{\text{X}}_{4} + 0.2945{\text{X}}_{5} + 0.4041{\text{X}}_{6} \\ & + \;0.3817{\text{X}}_{7} - 0.3322{\text{X}}_{8} + 0.2156{\text{X}}_{9} - 0.3331{\text{X}}_{10} + 0.4657{\text{X}}_{11} , \\ {\text{Y}}5 = & 0.3889{\text{X}}_{1} - 0.6895{\text{X}}_{2} + 0.144{\text{X}}_{3} + 0.1437{\text{X}}_{4} + 0.3889{\text{X}}_{5} - 0.2128{\text{X}}_{6} \\ & - \;0.1677{\text{X}}_{7} + 0.1894{\text{X}}_{8} - 0.1358{\text{X}}_{9} - 0.1427{\text{X}}_{10} + 0.1806{\text{X}}_{11} . \\ \end{aligned} $$

**Table 4:** Indices used to evaluate potato in conventional irrigation (CK) and regulated deficit drip irrigation treatments.

Treatment	Yield	Water use efficiency	Irrigation water use efficiency	Output value	Unilateral aquatic product value	Total sugar	Protein	Starch	Vitamin C	Potassium	Calcium
	(kg ha^−1^)	(kg m^−3^)	(kg m^−3^)	(yuan ha^−1^)	(yuan m^−3^)	(%)	(mg g^−1^)	(%)	(mg 100 g^−1^)	(mg kg^−1^)	(mg kg^−1^)
	X_1_	X_2_	X_3_	X_4_	X_5_	X_6_	X_7_	X_8_	X_9_	X_10_	X_11_
CK	46,033.23	7.82	18.09	59,843.21	26.48	0.58	2.17	36.06	12.76	5065.17	93.12
WD1	43,961.91	8.67	21.13	57,150.48	27.47	0.47	2.07	34.22	15.67	5106.18	87.26
WD2	41,016.67	8.18	18.87	53,321.67	31.93	0.42	1.77	32.59	16.95	5021.78	94.92
WD3	36,430.71	7.32	18.55	47,359.92	26.50	0.41	2.03	32.28	18.17	4968.96	96.24
WD4	42,262.25	8.18	20.19	54,940.91	39.97	0.52	2.18	32.7	17.08	4900.08	102.9
WD5	40,473.53	8.28	23.54	52,615.58	33.03	0.44	1.95	31.17	19.69	5016.32	95.12
WD6	37,461.29	7.88	19.59	48,699.67	31.21	0.42	1.69	27.77	19.69	4833.46	109.7
WD7	33,834.86	6.81	15.66	43,985.31	22.36	0.31	1.46	25.28	21.83	4695.38	123.29
WD8	37,599.61	8.21	22.79	48,879.49	32.90	0.58	2.17	36.06	12.76	4728.83	118.35

The irrigation treatments were conventional irrigation (CK) and mild or moderate water deficit during each of four stages of potato growth. Mild deficit was in treatments WD1 (seedling), WD2 (tuber initiation), WD3 (tuber bulking), and WD4 (starch accumulation); moderate deficit was in treatments WD5 (seedling), WD6 (tuber initiation), WD7 (tuber bulking), and WD8 (starch accumulation).

**Table 5:** Features of factor matrix extracted by five principal components.

Index	Factor				
	Y_1_	Y_2_	Y_3_	Y_4_	Y_5_
Yield	0.3456	0.0717	− 0.2953	0.2945	0.3889
Water use efficiency	0.2380	0.3620	− 0.4626	0.1369	− 0.6895
Irrigation water use efficiency	0.0638	0.6094	0.2021	− 0.0846	0.1440
Output value	0.0621	0.6100	0.2009	− 0.0831	0.1437
Unilateral aquatic product value	0.3465	0.0717	− 0.2953	0.2945	0.3889
Total sugar	0.3202	− 0.2455	0.2318	0.4041	− 0.2128
Protein	0.3131	0.0025	0.6051	0.3817	− 0.1677
Starch	0.3501	− 0.1024	0.2362	− 0.3322	0.1894
Vitamin C	− 0.3410	0.2000	0.2301	0.2156	− 0.1358
Potassium	0.3611	− 0.0627	0.0246	− 0.3331	− 0.1427
Calcium	− 0.3542	0.0229	− 0.0354	0.4657	0.1806

**Table 6:** Comprehensive index values and evaluation coefficients of potato in conventional irrigation (CK) and regulated deficit drip irrigation treatments.

Treatment	Y_1_	Y_2_	Y_3_	Y_4_	Y_5_	Overall rating
CK	2.90995	− 1.28064	− 0.48612	0.60484	0.62713	1.60239
WD1	3.60267	− 0.76356	− 0.93734	0.01499	− 0.42728	2.09771
WD2	1.18469	− 0.01058	0.46858	− 0.57993	0.14227	0.77424
WD3	− 0.78786	− 0.89969	0.00027	− 1.43324	− 0.04555	− 0.77183
WD4	0.44446	3.01387	0.15027	− 0.02647	0.73174	1.00346
WD5	1.55440	0.63227	0.86751	0.42978	− 0.80347	1.20040
WD6	− 1.27528	− 0.17980	1.17898	0.37273	− 0.07906	− 0.78804
WD7	− 4.14625	− 2.19086	0.00953	0.43179	0.26601	− 3.16771
WD8	− 3.48678	1.67900	− 1.25170	0.18551	− 0.41179	− 1.95061

The positive and negative values in the table indicate their position only relative to the average. The irrigation treatments were conventional irrigation (CK) and mild or moderate water deficit during each of four stages of potato growth. Mild deficit was in treatments WD1 (seedling), WD2 (tuber initiation), WD3 (tuber bulking), and WD4 (starch accumulation); moderate deficit was in treatments WD5 (seedling), WD6 (tuber initiation), WD7 (tuber bulking), and WD8 (starch accumulation).

According to the principal component analysis, the principal component values of deficit irrigation at different growth stages were ranked as WD1 > CK > WD5 > WD4 > WD2 > WD3 > WD6 > WD8 > WD7. In conclusion, WD1 was optimal in the valuation.

## Discussion

Regulated deficit irrigation abides by crop water-demand laws in each growth stage to induce water stress to different degrees, causing crop growth conditions to change so as to stabilize yield, save water, and adjust quality^25–28^. Deng et al.^29^ found that regulated deficit drip irrigation under film changes the hydrothermal environment of farmland soil, thereby affecting crop growth. In this study, the amount of water irrigated in drip irrigation affected the indices of plant height, stem diameter, tuber transverse diameter, tuber longitudinal diameter, and leaf area of potato. These indices all tended to decrease, and regulated deficit irrigation had a greater effect in the late growth stage than in the early growth stage. The explanation might be that the root system in the seedling stage was small and required less water. In addition, slight regulated deficit had a mild influence on potato growth. By contrast, the late growth stage is essential for nutrient production in potato and requires more water. Water stress inhibited the natural growth and development of potato, hindering tuber formation.

Photosynthesis is highly sensitive to water stress. Water deficit may hinder CO_2_ from entering leaves or affect the CO_2_ carboxylation ability of mesophyll cells, thereby inhibiting photosynthesis^30–32^. According to Reddy et al.^33^, water deficit can close the stomata in crop leaves; further reducing Gs and then Pn. In this study, in WD2 and WD6 and WD4 and WD8, the Pn decreased significantly in the tuber formation and starch accumulation stages. The decrease increased as the regulated deficit amount increased. Chai et al.^34^ found that as the regulated deficit degree gradually increases, the Gs decreases significantly, thereby lowering the Pn. This result is consistent with the conclusion of this study. A similar conclusion was also reached in a study of regulated deficit drip irrigation under film with *Isatis indigotica* in an oasis environment^35^. The result in this study might be explained by water deficit inhibiting the aboveground growth of potato, and as a result, both leaf area index and the transpiration rate decreased. In addition, with a water deficit in the soil, the partial or complete closure of stomata decreases the transpiration rate, thus leading to an overall decrease in net photosynthetic rate^36–38^. In this study, moderate water deficit led to a greater decrease in Gs, likely because the increase in water stress increased stomatal closure, which further reduced the transpiration rate.

In this study, regulated deficit irrigation decreased potato yield and output values to different extents, ranging from 4.50 to 26.50%, which reduced production benefits. Enciso et al.^39^ found that a moderate regulated deficit in the seedling and the mature stages can increase crop yields and economic benefits. The result is in contrast to those in this study, which may because of differences in regulated deficit amount, test conditions, and potato varieties. In this experiment, compared with the output in CK, mild (WD3) and moderate (WD7) regulated deficits in the tuber enlargement stage significantly decreased the output by 20.86% and 26.50%, respectively. Consistent with this study, Mustafa Ünlü et al.^40^, Hassan et al.^41^, Nagaz et al.^42^, and Im et al.^43^ also found that water deficit at the tuber enlargement stage reduces production by approximately 20% compared with that with a sufficient water supply. As tubers begin to divide and expand in the tuber enlargement stage, potatoes transition from the reproductive stage to the vegetative growth stage. Water deficit decreases potato transpiration and photosynthesis, and compensation and recovery with rehydration are difficult. As a result, yield is severely reduced, decreasing economic benefits. Compared with the output of potatoes in CK, the output a with mild regulated deficit in the seedling stage (WD1) decreased by only 4.50%, likely because root activity and absorption were low in the early growth stage. During the seedling stage, the water deficit likely promoted deep root penetration. With the irrigation deficit in the seedling stage, potato had a longer time to recover following the subsequent rehydration, and thus, water deficit had little effect on yield and economic benefits.

The highest WUE was in WD1, followed by that in WD5, WD4, and WD8, which increased by 10.87% (WD1), 5.84% (WD5), 4.60% (WD4), and 4.99% (WD8) compared with that in CK. The lowest WUE was in WD7 at only 6.81 kg m^−3^, which was a decrease of 12.92% compared with that in CK. The highest IWUE was in WD5, reaching 23.54 kg m^−3^, followed by that in WD8 (22.79 kg m^−3^), which increased by 30.13% and 25.98%, respectively, compared with that in CK. The lowest IWUE was in WD7 (15.66 kg m^−3^), which was a decrease of 13.45% compared with that in CK. The IWUE of the other treatments increased to different degrees, with increases ranging from 2.52 to 30.13%. Therefore, moderate water deficit at the seedling and starch accumulation stages helped to improve the WUE and IWUE of potato, consistent with the findings of Li et al.^44^ and Liuyang et al.^45^. However, an unreasonable water deficit can cause significant yield reduction and thus reduce output and benefits.

While maintaining yield, timely and moderate water deficit can increase WUE and the quality of products^46–48^. In this study, a moderate regulated deficit increased protein, starch, vitamin C, potassium, and calcium contents in potato. Mild regulated deficit in the seedling stage increased starch and potassium contents without reducing total sugar content. By comparison, moderate regulated deficit increased protein, vitamin C, and calcium contents. Regulated deficit irrigation in the other growth stages did not lead to the accumulation of total sugar, protein, starch, and potassium. Guizani et al.^49^ found that water deficit in the seedling stage can improve potato quality, which is consistent with the conclusion in this study. However, Zhang^50^ found that a regulated deficit had no significant effect on starch content during potato growth stages. In contrast, the results of in this study showed that a regulated deficit significantly reduced potato starch content. The inconsistency between studies might be due to differences in factors such as soil type and potato variety. Because there are few comprehensive reports on how the amount of regulated deficit and the stages in which the deficit occurs affect potato yield and quality, additional experiments are needed to explore the effects of regulated deficit irrigation on yield and quality with different varieties and in different regions.

## Materials and methods

### Description of experimental site and soil

**Figure 4:**
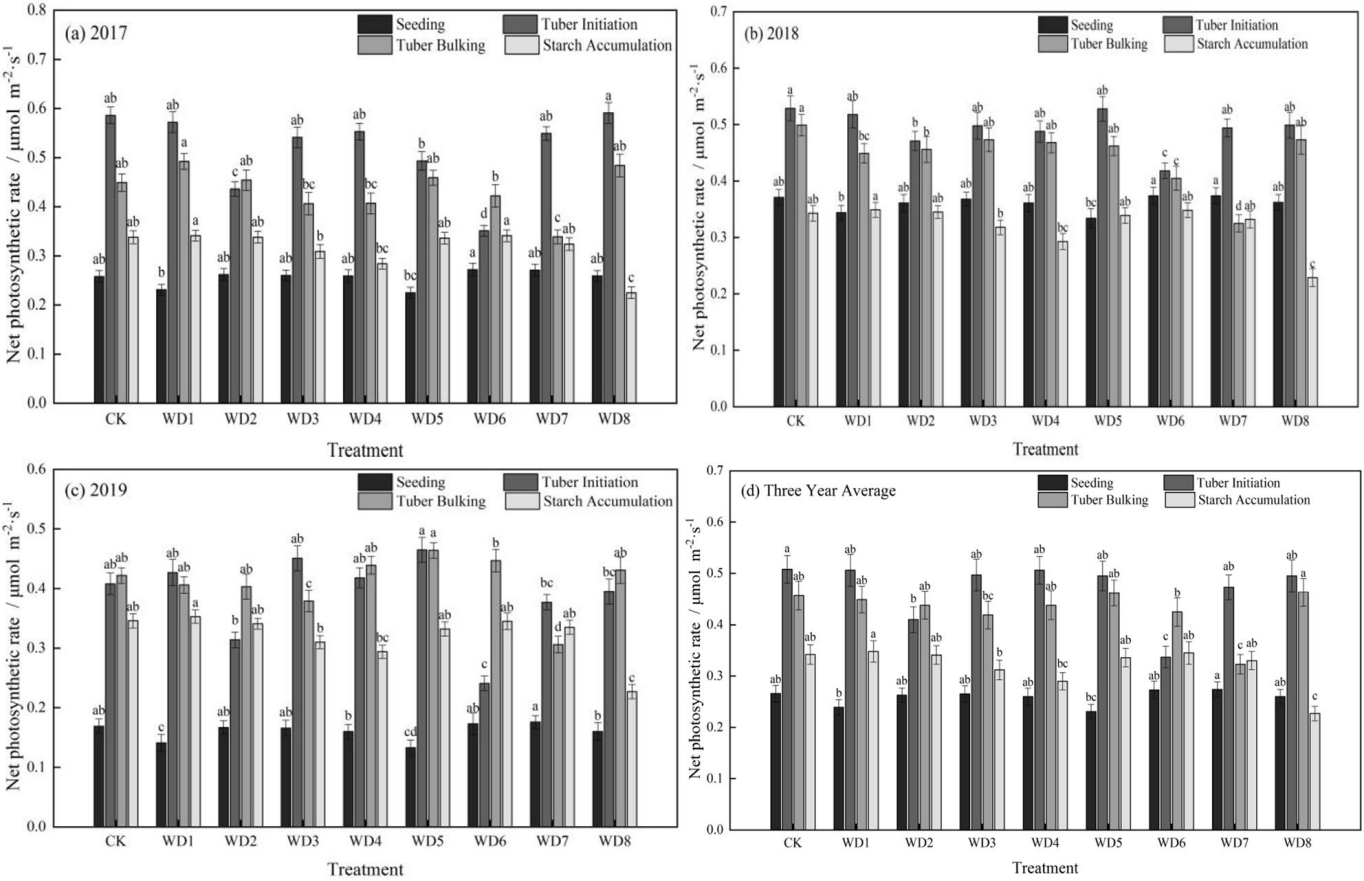
Rainfall and temperature in three potato seasons (2017, 2018, 2019) at the *Yimin* Irrigation Experimental Station, *Hongshui River Administrative Office*, Minle County, *Gansu* Province, *China*.

The experiment was conducted at the *Yimin* Irrigation Experimental Station (38° 39′ N, 100° 43′ E, approximately 1970 m) of the *Hongshui* River Administrative Office, Minle County, *Gansu* Province, *China*, from March to September in 2017, 2018, and 2019. The area has a typical continental arid climate, with abundant sunshine and large differences between day and night temperatures, which are conditions conducive to photosynthesis, nutrient accumulation, and yield formation in crops. The average annual temperature is 6.0 °C. The thermal integral exceeding 0 °C is 3,500 °C day, whereas the thermal integral over 10 °C is 2985 °C day”. The average annual sunshine time is 3000 h, and the average frost-free period is 136 days. According to meteorological data from 2000 to 2018, the average annual rainfall in the area is 328 mm, and the evaporation is 1900 mm. Soil in the experimental site is light loam with medium fertility and a pH of 7.22. The field capacity of tilled soil is 24.0%, the wilting point of the soil is 8.2%, and the soil bulk density is 1.48 t/m^3^. The 0 to 20 cm of topsoil contained 12.8 g/kg organic matter, 63.5 mg/kg alkali-hydrolyzable nitrogen, 13.1 mg/kg available phosphorus, and 192.7 mg/kg available potassium. The salinization effect was mild because of the deep source of groundwater. The rainfall and temperature for the three potato seasons are shown in Fig. 4. The total seasonal rainfall was 219.25 mm in 2017, 222.2 mm in 2018, and 305.13 mm in 2019. In 2017, 2018, and 2019, the highest precipitation was from May to September (Fig. 4). In all years, the highest precipitation was from May to September. The experimental research and field studies on plants, including the collection of plant material were in accordance with the Irrigation Experiment Standard (SL 13-2004) issued by the Water Industry Standards of the People’s Republic of China.

### Experimental design and drip irrigation systems

**Figure 5:**
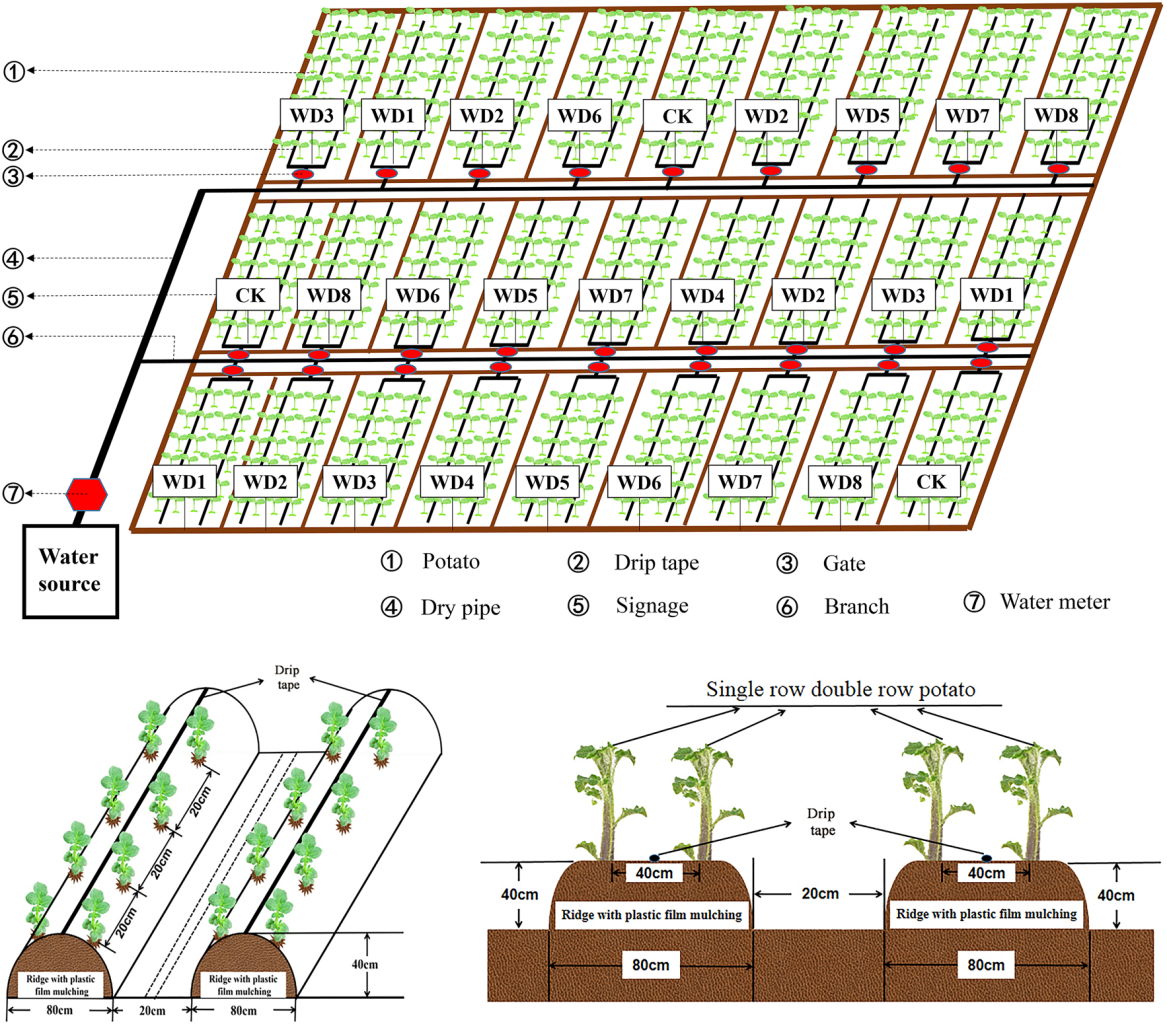
Cultivation of potatoes with regulated deficit drip irrigation on ridges under plastic film mulching.

The potato variety ‘Qingshu 168’ was provided by the *Qinghai* Agricultural Science Research Institute of China *Qinghai* Agricultural Research Institute. In 2017, potatoes were sown on April 17 and harvested on September 27; in 2018, they were sown on April 11 and harvested on September 28; and in 2019, they were sown on April 14 and harvested on October 9. The row spacing was 40 cm, and the plant spacing was 20 cm (Fig. 5). A white plastic film (140 cm wide, 0.01 mm-thick; *China Dongguan Shuotai Industrial* Co., Ltd.) covered two rows of potatoes with a planting density of 77,000 plants/ha. Drip irrigation was applied under the film with the irrigation pipe placed between two rows. Each treatment and control were repeated three times, and 140 potato plants were sown in each test plot. Each section covered 33.6 m^2^ (7 m × 4.8 m). There were two levels of water deficit: mild with soil moisture at 55–65% of field capacity and moderate with soil moisture at 45–55% of field capacity. The soil moisture with conventional irrigation (CK) was 65–75% of field capacity. Each level of deficit was applied in each of four growth stages of potato: seedling, tuber initiation, tuber bulking, and starch accumulation stages. Thus, there were eight total treatments: WD1: mild, seedling; WD2: mild, tuber initiation; WD3: mild, tuber bulking; WD4: mild, starch accumulation; WD5: moderate, seedling; WD6: moderate, tuber initiation; WD7: tuber bulking; WD8: starch accumulation (Table 7).

**Table 7:** Soil water content (% field capacity) in conventional irrigation and regulated deficit drip irrigation treatments during potato growth.

Treatment	Deficit	Seedling (%)	Tuber initiation (%)	Tuber bulking (%)	Starch accumulation (%)
WD1	Slight	55–65	65–75	65–75	65–75
WD2	Slight	65–75	55–65	65–75	65–75
WD3	Slight	65–75	65–75	55–65	65–75
WD4	Slight	65–75	65–75	65–75	55–65
WD5	Medium	45–55	65–75	65–75	65–75
WD6	Medium	65–75	45–55	65–75	65–75
WD7	Medium	65–75	65–75	45–55	65–75
WD8	Medium	65–75	65–75	65–75	45–55
CK	Conventional	65–75	65–75	65–75	65–75

The treatments were two levels of water deficit that occurred in each of four growth stages.

The data in the table show the percentage of soil mass water content in field water holdup under different experimental treatments. "Slight" means that the soil water deficit level was controlled at a “Mild” level with soil water content maintained at 55–65% of field capacity during certain plant growth stage in the treatment while it was maintained at 65–75% of field capacity at the other plant growth stages in the same treatment. Similarly, "Medium" means that the soil water deficit level was controlled at a “Moderate” level with soil water content maintained at 45–55% of field capacity during certain plant growth stages in the treatment while it was maintained at 65–75% of field capacity at the other plant growth stages in the same treatment.

### Agronomic practices

To ensure crop yield, the experimental section was tilled to 30 cm 10 days before sowing. Weeds were cleared manually. Diammonium phosphate (18% nitrogen and 46% P_2_O_5_) at 400 kg/ha and western compound fertilizer (15% nitrogen, 15% P_2_O_5_, and 15% K_2_O) at 750 kg/ha were applied as base fertilizers one time at sowing.

## Measurements and calculations

### Potato growth indices

After potatoes entered the mature stage, 20 potato plants were randomly selected from each section. Plant height was measured with a ruler that was accurate to 0.1 cm, and stem diameter was measured with vernier calipers that were accurate to 0.01 mm.

### Physiological indices

Photosynthetic indicators were measured starting on the 5th day after the regulated deficit treatment in each reproductive period. Three separate measurements were taken on 3 days during each reproductive period when the weather was clear, and the results were the mean values of three measurements. Each measurement was conducted using a LI-6400 portable photosynthesis system (LI-COR, USA) from 9:30 to 11:00 a.m. Photosynthetic indicators measured included net photosynthetic rate (Pn), stomatal conductance (Gs), and transpiration rate (Tr).

### Yield and tuber morphological properties

After potatoes ripened, the potatoes in each section were harvested and measured. There were three plots in each experiment, and the average value of the three plots was taken for analysis in each experiment. In other words, the average value of three replicate plots was the output of each treatment. An electronic scale (accuracy to 0.01 g) was used to weigh the potatoes, and the yield was converted to kg/ha. In addition, 20 potato plants were harvested separately in each section, and the tubers were cut off and washed. The transverse and longitudinal diameters of the tubers were measured with vernier calipers that were accurate to 0.01 mm. The average value of each treatment was determined.

### Water use efficiency and irrigation water use efficiency

Water use efficiency (WUE) and irrigation water use efficiency (IWUE) were calculated using the following formulas^50^:

$$ \begin{aligned} & {\text{WUE }} = {\text{ Y}}/{\text{ETa}}, \\ & {\text{IWUE }} = {\text{ Y}}/{\text{I}}, \\ \end{aligned} $$

where WUE (kg/m^3^) and IWUE (kg/m^3^) are the water use efficiency and the irrigated water use efficiency, respectively, in all growth stages; Y (kg/ha) is the yield per unit area; ETa (m^3^/ha) is the actual water consumption per unit area in all growth stages; and I (m^3^/ha) is the irrigation amount per unit area in all growth stages.

### Irrigation volume

The experiment used PVC pipe water delivery and drip irrigation under the film to irrigate water. All gate valves and water meters were installed in each treatment area, and the corresponding irrigation volume was quantitatively controlled by the water meter. When the soil water content dropped to the lower limit of the design value, irrigation was should be carried out in time, and the required irrigation amount was calculated by the irrigation quota formula:^51^

$$ {\text{M}} = {1}0\rho b{\text{H}}(\beta_{i} - \beta_{j} ), $$

where M (mm) is the irrigation volume, *ρb*(g/cm^3^)is the soil bulk density of the planned wet layer. H (cm) is the depth of the soil plan wet layer, β_i_ is the target moisture content (field water holding capacity multiplied by the upper limit of the design target relative moisture content), and β_j_ is the soil moisture content before irrigation.

### Leaf area index

In the four growth periods of potatoes, for each treatment plot, three uniformly growing potatoes, were randomly selected. All the leaves, were cut from each potato in order to measure the maximum length and maximum width of the leaves and calculate the area of all the leaves. The total leaf area of a single plant was derived from the sum of all leaf areas, the leaf area was corrected with a coefficient of 0.76, and the average leaf area was finally taken^52^.

$$ \begin{aligned} {\text{Leaf area}}\left( {{\text{cm}}^{{2}} } \right) = & {\text{ Maximum leaf length}}\left( {{\text{cm}}} \right) \, \\ & \times \;{\text{Maximum leaf width}}\left( {{\text{cm}}} \right) \times 0.{76}. \\ {\text{Leaf area index}} = & \left( {{\text{Total leaf area per plant }} \times {\text{ total number of potato plants in the plot}}} \right) \\ & \div \left( {\text{plot area}} \right). \\ \end{aligned} $$

### Soil moisture content

The test mainly followed the traditional soil drilling and soil drying weighing method to determine the soil moisture in each treatment plot. According to previous research results, the root activity range of potato is mainly concentrated in the 0–40 cm of the soil layer. Therefore, the soil was sampled every 7 days during the whole growth period of the potato, and the sampled soil depth was 80 cm, which was divided into five parts: 0–10 cm, 10–20 cm, 20–40 cm, 40–60 cm, and 60–80 cm. The soil water content (SWC) is calculated as follows:^51^

$$ {\text{SWC }}\left( \% \right) \, = \, \left( {{\text{W1}}{-\!\!-}{\text{W2}}} \right) \, / \, \left( {{\text{W2}}{-\!\!-}{\text{W3}}} \right) \, \times {1}00\% \, , $$

where SWC (%) is the soil moisture content; W1(g) is the total mass of the fresh soil sample and the aluminum box; W2(g) is the total mass of the dried soil sample and the aluminum box; and W3(g) is the mass of the empty aluminum box. The detailed soil moisture data of the 3-year field experiment are shown in supplementary Table S1, Table S2 and Table S3.

### Calculation of evapotranspiration

Evapotranspiration was calculated using the water balance method:^53^

$$ ET_{{{\text{I}} - {\text{II}}}} = 10\sum\limits_{i = 1}^{n} {ri} Hi(Wi_{{\text{I}}} - Wi_{{{\text{II}}}} ) + M + P + K - D $$

where *ET*_I–II_ (mm) is the water consumption in the potato stage; *i* is the serial number of the soil layer; *n* is the total number of soil layers; *r*_*i*_ (g·cm^−3^) is the dry bulk density of the *i*th layer of soil; *H*_*i*_ (cm) is the thickness of the *i*th layer of soil; $Wi_{{\text{I}}}$, and $Wi_{{{\text{II}}}}$ respectively are the mass water content of the ith soil at the beginning and end of the growth period, respectively, %; *M* (mm) is the amount of irrigation water during the growth period; *P* (mm) is the rainfall during the growth period; and *K* (mm) is the amount of groundwater replenishment in the growth period. The groundwater depth in the test area is greater than 20 m, so *K* = 0. *D* (mm) is the drainage volume within the stage, and there is no runoff drainage in the test area, so *D* = 0.

### Quality

The Coomassie Brilliant Blue G-250 method was used to determine protein content, and anthrone colorimetry was used to determine total sugar content. The content of starch was determined by enzymatic hydrolysis, vitamin C by 2, 6-dichloroindophenol titration, potassium by the potassium tetraphenylborate gravimetric method, and calcium by the gravimetric titration method.

### Statistical analyses

Excel 2017 (Microsoft 365) was used to perform calculations, and Duncan’s multiple comparison method in SPASS 19.0 (Stanford University) software was used to determine significant differences between means. Origin 8.0 (Origin lab) was used prepare the diagrams of average values. All analyses were performed using 3-year (2017, 2018 and 2019) averages.

## Conclusions

In the *Hexi* Corridor Oasis Irrigation Area, due to low rainfall, high evaporation intensity, water shortage, serious irrigation water waste and other factors, the development of local agriculture has been severely restricted. To minimize these negative impacts, we tested a novel planting method: regulated deficit drip irrigation under film mulching. The experiment found that different levels of water deficit in different growth periods of potatoes will affect the growth and development of their potatoes, and the degree of impact will continue to increase as the growth period advances. The analysis showed that the best water regulation scheme was mild water stress in the seedling stage. However, there was no significant difference in plant water content among potato treatments after harvest, the values of the water status of the plant is 74.10–79.70%. Therefore, the relative water content in soil should be maintained between 55 and 65% in the seedling stage, whereas in the other growth stages, it should range from 65 to 75%. The irrigation method proposed in this study can simultaneously stabilize output and maintain good quality, which is significant for yield improvement, water conservation, quality adjustment, and industrialization of potatoes cultivated in the *Hexi* Corridor Oasis Irrigation Area.

## Supplementary Information

Supplementary Information.
